# Task cue transparency shapes cognitive and visual demands in task preparation

**DOI:** 10.1007/s10339-026-01331-x

**Published:** 2026-02-06

**Authors:** Alexander Berger, Markus Kiefer

**Affiliations:** Department of Psychiatry, Section for Cognitive Electrophysiology, Ulm University, Leimgrubenweg 12, 89075 Ulm, Germany

**Keywords:** Task cue, Task cue transparency, Proactive control, EEG, ERP, Wavelet

## Abstract

Task cues are often used to study task preparation. Participants are thought to activate cued task sets in advance to facilitate later task performance. Earlier studies showed that task performance was improved, if the relation of cue and decision categories of the task was more transparent, suggesting a facilitated task preparation process with more transparent cues. However, no previous study directly tested how processing differs between cues with varying transparency in the cue interval itself. To this end, we analyzed cue-locked event-related potentials (ERPs) and oscillatory activity of the electroencephalogram (EEG) for four different cue types with varying cue transparency. An increased cue-locked positivity ERP component indicated the largest preparation demands for arbitrary symbol string cues, which lacked any apparent relation with the cued task. Moreover, visual demands reflected by an early positive deflection and task set reconfiguration demands reflected by theta oscillations were increased for both word cues (decision categories used as cues) and symbol string cues, indicating that cues with a higher visual complexity pose additional demands on visual and cognitive cue processing. In contrast, these electrophysiological correlates of preparation demands were lowest for letter cues. Hence, if one wants to facilitate cue-induced task set activation, simple letter cues appear beneficial. In conclusion, task cue transparency influenced electrophysiological correlates related to the preparatory demands required for task set retrieval and should therefore be taken into account when studying task preparation.

## Introduction

In situations requiring individuals to perform different tasks, often so-called task cues are used to inform about the nature of the to-be-performed task. Task cues vary strongly in format (e.g., symbols, word, colored frames) but are always intended to activate a task set, which can be defined as the cognitive configuration required for task application (Monsell 2003; Rogers and Monsell 1995). Previous work showed transparent cues with a more direct link to the cued task set to facilitate task preparation (Arbuthnott and Woodward 2002; Gade and Koch 2014; Gade and Steinhauser 2020; Grange and Houghton 2010), for a review see Jost et al. (2013). However, these studies only collected behavioral data, which can only reflect the consequences of task preparation (i.e., response time in the cued task), but not the preparation process itself. The present study therefore aimed to investigate how task cue transparency affects task preparation more directly by assessing electrophysiological activity during the cue interval.

### Role of task cues for task preparation

Task cues can be presented simultaneously with the stimulus or before stimulus onset, with the latter one enabling preparation for a task (for reviews, see: Jost et al. 2013; Kiesel et al. 2010; Koch et al. 2018). The role of task cues was often studied in task switching, where switch costs (difference task switch – task repetition) are reduced with a longer time interval available for cue processing (cue-target interval, CTI). Reduced switch costs with longer preparation time indicate that participants use the cue to prepare for the upcoming task, i.e., activate the relevant task set in advance (Jost et al. 2013; Kiesel et al. 2010; Koch et al. 2018). Such advance preparation is also called *proactive* control, and is usually separated from *reactive* control processes occurring after stimulus onset, i.e., during task processing (Jamadar et al. 2015). Effects of proactive control processes elicited by task cues were however not only studied in task switching contexts, but also in other designs, like in the context of a modulation of unconscious processing by task sets.

### Modulation of unconscious processing by executed tasks and task cues

The role of task cues was also examined in the context of the attentional sensitization model of unconscious cognition, which postulates that activated task sets influence subsequent unconscious processing through attentional mechanisms (Kiefer 2012; Kiefer and Martens 2010). For instance, the execution of a semantic task is supposed to amplify subsequent unconscious semantic priming, a finding being demonstrated in several studies (Adams and Kiefer 2012; Kiefer 2019; Kiefer and Martens 2010; Martens et al. 2011; Ulrich et al. 2014). Motivated by these findings, in two studies we investigated whether also task sets triggered by mere task cue presentation lead to a similar modulation of unconscious semantic priming (Berger et al. 2022; Kiefer et al. 2019).

Both studies incorporated two trial types: (1) *Induction task trials*: here, the influence of executed tasks on priming was assessed by probing the effect of an induction task on subsequent unconscious semantic priming in a masked primed lexical decision task (LDT). (2) *Task cue-only trials*: here, the induction task cue was directly followed by the LDT, omitting the stimulus on which the cued task set needed to be applied, therefore testing the influence of mere task preparation on masked priming. Both trial types incorporated semantic or perceptual task sets, which should either amplify (semantic task set) or attenuate (perceptual task set) subsequent semantic priming. In general, in these studies masked semantic priming was attenuated following a semantic task cue-only trial, indicating that merely cued – but not executed – task sets become inhibited, probably to facilitate switching to the subsequent LDT (Berger et al. 2022; Kiefer et al. 2019), see also Berger et al. (2024). Motivated by hints that task set inhibition effects following task cue-only trials depended on task cue transparency in the first study (Kiefer et al. 2019), we manipulated the relation between cue and response categories more explicitly, to investigate how the suppression of task sets in task cue-only trials depends on such properties (Berger et al. 2022). Cues were verbal and the relation of cue and response categories was varied to different degrees in four participant groups, see Table 1.

**Table 1:** Different cue types used in Berger et al. (2022) and the present study

Cue type	Task		Note
	rund/länglich	belebt/unbelebt	“rund/länglich” are the response categories of the perceptual task (engl. round/elongated), “belebt/unbelebt” the response categories of the semantic task (engl. living/non-living).
rund/belebt	rund	belebt	
R/B (comp)	R	B	The abbreviations comp (compatible) and incomp (incompatible) were added to facilitate comparison of cue types.
B/R (incomp)	B	R	
$$$$$/§§§§§	$$$$$	§§§§§	In one group of participants, the symbol string “$$$$$” cued the semantic task and the symbol string “§§§§§” the perceptual task. This assignment was reversed in the other participant group. Assignment to both groups was counter-balanced across participants, and data was aggregated across groups for analysis.

In detail, task cues were word cues identical to the decision categories (rund/belebt – fully transparent cues), were letter cues matching with the first letter of the decision categories (R/B [comp] – transparent cues), showed the first letter of the decision category of the alternative task (B/R [incomp], i.e., letter cues matching with the first letter of the other response category, therefore termed incompatible cues – semi-transparent cues) or were a symbol string which lacked any apparent relation with the cued task ($$$$$/§§§§§ – arbitrary cues). Note that while for both cue types B/R (incomp) and $$$$$/§§§§§, cues did not match with the associated response category, retrieval of task sets nevertheless could be (somehow) facilitated for the letter cues, as the reversed assignment may serve in terms of a mediator facilitating task set retrieval (e.g., cue “B” → mediator “use opposing response category” → response category “rund/länglich”), while for the symbol cues no such mediator could be used (cf. Logan and Schneider 2006).

In this study, cue type did not affect the modulation of masked semantic priming in task cue-only trials, thus probably all cue types activated task sets to a similar degree, triggering an inhibition of cued task sets when the LDT instead of the cued task followed (Berger et al. 2022). However, drift-diffusion model analyses (Ratcliff and McKoon 2008; Voss et al. 2013) showed the different cue types to influence general task performance, and despite a similar level of task set activation (until task cue offset), the engagement into cue processing might therefore have differed between cue types. This could be tested by means of electro-encephalographic (EEG) data recorded in the cue interval. Hence, below we will outline research addressing how task preparation demands may be reflected by recordings of neural activity.

### Neural correlates of proactive control induced by task cues

Most previous research on cognitive demands for task preparation (proactive control) used task switching paradigms. While the design of the present study differed from a typical task switching study regarding the trial sequence (see the Method), both designs involve the presentation of task cues, which indicate an upcoming task and thus should trigger task preparation. Therefore, neural activity recorded in the cue interval in task switching studies should reflect task preparation demands similarly to the present study.

These task switching studies often investigated EEG activity (Karayanidis et al. 2010), as the high temporal resolution of the EEG on the time scale of milliseconds enables separating proactive (cue processing) from reactive processing (stimulus/task processing). The most prominent marker of cognitive demands for task preparation is the so-called switch positivity (for a review, see: Jamadar et al. 2015), a larger cue-locked positive event-related potential (ERP) component in switch compared to repeat trials (Cunillera et al. 2012; Kieffaber and Hetrick 2005). However, this cue-locked positivity component is not only sensitive to task switches vs. repetitions. For example, a cue-locked positivity was increased for mixed-tasks blocks compared to single-task blocks (Jost et al. 2008), was larger for informative compared to non-informative cues (Karayanidis et al. 2009) and depended on the amount of interference in the cued task (Sinai et al. 2007). Accordingly, it may reflect a more general marker of task preparation demands (Jamadar et al. 2015), with cues associated with more demanding cognitive processes eliciting a larger cue-locked positivity.

Besides ERPs, analyses of oscillatory activity in the EEG may reveal further insights about task cue processing. Oscillations are thought to be a key determinant of functional communication between brain regions required for complex cognitive processing (for reviews, see: Başar et al. 2001; Le Van Quyen and Bragin 2007). Recent research indicated the theta, alpha and beta band to be related to proactive control. Theta oscillations, which are supposed to represent a general index of cognitive control (Cavanagh and Frank 2014), were consistently increased following task cues indicating a task switch compared to a task repetition (Cooper et al. 2017; Cunillera et al. 2012; López et al. 2019; Mansfield et al. 2012). For alpha and beta oscillations, results are more scarce: One study reported decreased alpha / beta oscillation in switch trials (Cunillera et al. 2012), while another one contrasted switch and repeat cues with non-informative cues, and could observe increased alpha / beta power only for repeat cues (Mansfield et al. 2012). To sum up, theta, alpha and beta oscillations were modulated in preparation for a task switch, presumably indexing demands for task cue processing, while the theta band showed the most consistent modulations.

### Overview of the present study

In the present study, we re-analyzed EEG data collected in Berger et al. (2022), incorporating four different cue types with varying task cue transparency. Note that to the best of our knowledge, no previous study compared preparatory demands as measured by EEG between different cue types. Consequently, the present analyses should give insights beyond previous research regarding the question, which types of cues may be advisable if a researcher wants to facilitate the cue-induced task preparation process.

Proactive control processes were investigated by means of cue-locked event-related as well as oscillatory activity. As our experimental paradigm remarkably differed from typical task switching experiments, it is unclear whether the same ERP components and frequency bands reflect task preparation. Thus, we incorporated a two-step analysis procedure. First, we compared cue-locked ERPs and wavelets between all four cue types using cluster-based permutation testing (CBPT), to identify ERP components and frequency bands reflecting task preparation differences between cue types. CBPT controls for the false-discovery rate and is therefore suited for comparing EEG activity without a priori defined regions and/or time intervals of interest (Maris and Oostenveld 2007). Likewise, beyond components identified by previous task switching studies, differences in early ERP components like the N1 (Clark and Hillyard 1996; Vogel and Luck 2000), P1 (Clark and Hillyard 1996; Mangun and Hillyard 1991), N2 and P2 (Luck and Hillyard 1994; Potts 2004), which might reflect visual and early attentional processes, can be detected by the present analyses as well. In a second step, we extracted activity from the observed significant clusters, to further identify how EEG activity specifically differed between the four cue types, i.e., which differences between particular cue types might have caused the observed significant cluster in the CBPT analysis.

In line with previous observations in task switching, we expected a cue-locked positivity component (similar to the switch positivity) to be increased for more “difficult” cues, reflecting larger demands for proactive control, if task cues are less transparent. Similarly, we hypothesized theta power to be increased for such less transparent cues. Accordingly, these analyses should reflect a monotonic decrease in preparation demands (reduced cue-locked positivity and theta power) with increasing transparency of task cues ($$$$$/§§§§§ → B/R [incomp] → R/B [comp] → rund/belebt).

## Methods

The present work analyzed electrophysiological brain activity collected in a previous study (Berger et al. 2022). The study included four participants groups, which differed only according to the task cues used for cuing the induction tasks (see Table 1). While the behavioral analyses of both experiments were pre-registered (https://osf.io/gahxy, https://osf.io/ahk35), the analyses reported in the present work were performed post-hoc to further elucidate mechanisms underlying the obtained behavioral result pattern.

### Participants

We collected a total number of 115 German, right-handed (Oldfield 1971) participants. To remain consistent with the behavioral analysis of the present data, fourteen participants were excluded according to the same reasons as in Berger et al. (2022): above-chance performance in the masked prime identification task (*N* = 7), mean RT exceeding +- 2SD of the sample mean either in the lexical decision task or in the induction tasks (*N* = 5), as well as an error in the experimental software and while recording the EEG signal (each *N* = 1). The final sample consisted of 101 participants with a mean age of 22.9 (SD = 3.2). 71 participants were female. The number of analyzed participants was distributed across the different cue types accordingly: rund/belebt: *N* = 24, R/B (comp): *N* = 26, B/R (incomp): *N* = 27, $$$$$/§§§§§: *N* = 24.

### Paradigm

**Fig. 1:**
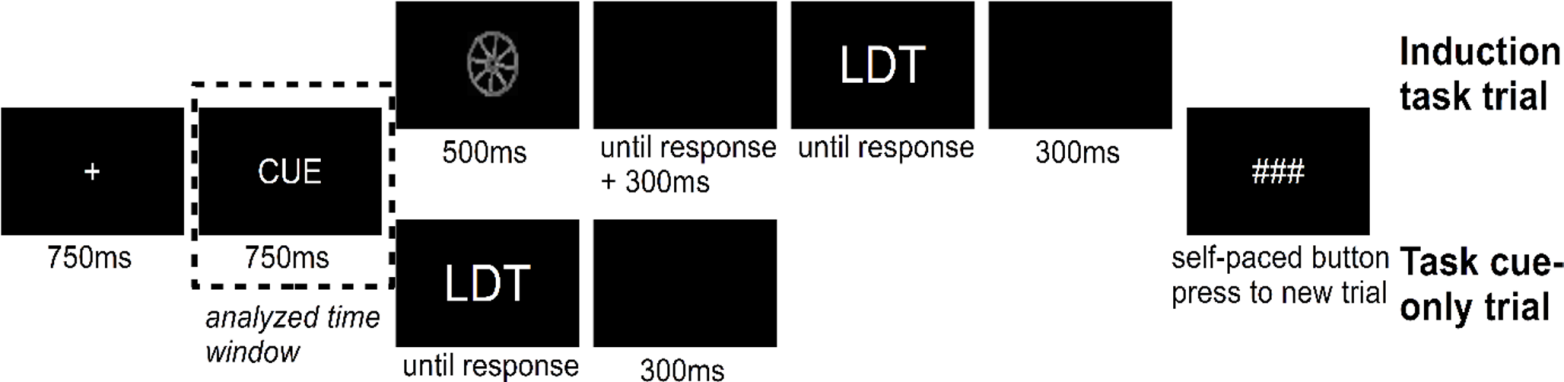
Graphical depiction of the trial sequence. There were two types of trials, which were collapsed for statistical analysis. In induction task trials, the task cue was followed by an induction task stimulus, on which the cued task had to be executed. Afterwards, a masked primed lexical decision task (LDT) was presented, including a forward mask (100ms), the prime word (33.5ms), a backward mask (33.5ms) and the LDT target (until response). After a 300ms blank interval, the next trial could be initiated with a self-paced button press. In task cue-only trials, the LDT immediately followed the task cue, omitting the induction task stimulus on which the cued task had to be applied. Only the task cue interval was analyzed. “CUE” is a placeholder for the four different cue types used in this study

This paragraph will only summarize the sequence of both trial types used in the present study. For information about stimulus material, practice and prime identification task please refer to Berger et al. (2022). This information is of no particular relevance for the present article focusing on cue processing and therefore omitted. Participants performed an experiment consisting of two trial types, which were presented intermixed and in random order: induction task trials and task cue-only trials (for a graphical depiction, see Fig. 1). Both trial types started with a fixation cross (750ms), followed by a task cue (750ms), indicating either a semantic (living/non-living decision) or a perceptual (round/elongated decision) task. In induction task trials, a picture was shown (500ms) after cue offset, on which the cued classification task had to be applied. After the response and a 300ms blank, a masked primed lexical decision task (LDT) followed, consisting of a forward mask (100ms), the prime word (33.5ms), a backward mask (33.5ms) and the LDT target. The LDT target remained on the screen until a response was given and had to be classified as German word or pronounceable, but meaningless pseudoword. After a 300ms blank, at the end of each trial, three hash marks were shown and participants could initiate the next trial with a self-paced button press. In task cue-only trials, the LDT immediately followed the offset of the task cue, omitting the induction task. The sequence was otherwise identical.

The experiment consisted of 640 trials, evenly distributed across the two trial types (induction task trials and task cue-only trials). In half of each trial type a semantic, in the other half a perceptual task was cued (each *n* = 160). While the material of the LDT is of no particular relevance for the present study, the LDT conditions (prime and target semantically related, prime and target unrelated, pseudoword target) were evenly balanced across the task set and trial type conditions.

### EEG recording and preprocessing

The EEG was recorded with a BrainAmp amplifier using BrainVision Recorder software (BrainProducts, Gilching, Germany). The setup consisted of 64 equidistant sintered Ag/AgCl electrodes. Grounding electrode and reference electrode were placed between AFz and Fz, as well as FCz and Cz, respectively. Sampling rate was 500 Hz and impedances were kept below 5 kΩ.

Preprocessing and analysis of EEG data was performed using FieldTrip, version 20201214 (Oostenveld et al. 2011). Continuous data was low-pass (30 Hz), high-pass (0.1 Hz) and DFT-filtered (50, 100, 150 Hz). Afterwards, data was segmented from 1500ms before cue onset to 1500ms after cue offset for induction task trials and 1500ms before cue onset to 1667ms after cue offset for task cue-only trials.^1^ Thus, the segment length before cue onset and after cue offset was (roughly) equal. If this data segmentation procedure resulted in a trial overlapping with the previous one, this trial was excluded, separately for induction task and task cue-only trials. Trials with an RT outlier were identified as trials with an RT exceeding +-2SD of the respective individuals mean RT and excluded from all subsequent analyses (Berger and Kiefer 2021, 2023). Bad channels were identified by visual inspection and replaced by the mean of all surrounding channels. Subsequently, ocular artifacts were removed via PCA and the data was re-referenced to an average reference. Finally, an automatic artifact rejection was performed using built-in FieldTrip functions with a z-value of 30. In contrast to the analysis of behavioral data reported in Berger et al. (2022), which analyzed the modulation of priming only in trials including a word target in the LDT, for the present analysis we included all trials regardless of the LDT condition. As the cue was presented before the LDT, the LDT condition therefore could not affect the processing of task cues.

For ERP analyses, artifact-free trials with a correct response were averaged. We excluded trials with a subsequent incorrect response from analysis, as an incorrect response could be the consequence of an error while activating / preparing the relevant task set. Considering the analysis of wavelets, artifact-free trials with a correct response were transformed via a Morlet wavelet transformation to the frequency space in the frequency range of 1 to 30 Hz in steps of 0.244 Hz with a temporal resolution of 10 ms. Number of wavelet cycles / Morlet parameter was set to 7 (FieldTrip default) and the power spectrum was returned. For analyses of ERPs, the data was baseline corrected for a time window of -300ms and − 150ms relative to cue onset, i.e., the window started 450ms after onset of the fixation cross and ended 150ms before offset of the fixation cross and should therefore contain no task-related activity. The baseline interval was chosen to be in line with previous studies investigating cue processing by means of ERPs and oscillatory activity, whose baseline periods included time intervals spanning from 300ms to 50ms previous to the cue (Cooper et al. 2017; Cunillera et al. 2012; Mansfield et al. 2012). For the analysis of wavelets, we shifted the baseline an additional 100ms away from cue onset, to avoid cue-related oscillatory activity to smear into the baseline period.^2^ Thus, the baseline period for the wavelet analysis was − 400ms to -250ms. Regarding the baseline period for the wavelet analysis, a relative baseline correction approach was chosen. Consequently, obtained power values reflected the increase / decrease relative to the baseline window. These pre-processing steps resulted in an average number of 462.3 (SD = 54.8) trials per participant included in the statistical analysis of ERP as well as wavelet data.

### Statistical analyses

Significance testing was performed with cluster-based permutation tests (CBPT, Maris and Oostenveld 2007; Sassenhagen and Draschkow 2019), which control for the false-discovery rate and therefore are well suited for high-dimensional EEG data (condition × channels × time [× frequency]). Clusters were formed according to a neighborhood structure defining each surrounding electrode as neighbor. To restrict clusters to local maxima, only time points with at least three neighboring significant channels were considered for cluster formation. Sample alpha was set to α = 0.05 and only clusters with a p-value < 0.05 were considered significant. Significance was tested against 3000 random permutations, and the analyzed time window reached from cue onset (0ms) to cue offset (750ms). To account for the different types of cues in this study, we contrasted cue types using an independent F-test on the sample level, reflecting a one-way ANOVA with the factor cue type with four levels. These analyses served to identify relevant ERP components and frequency bands reflecting preparatory task demands differing between (any of) the four different cue types.

If these analyses revealed significant clusters, we extracted for each participant the mean voltage averaged across all time points and electrodes included in an ERP cluster, and respectively the mean power change relative to baseline averaged across time points, electrodes and frequencies included in a wavelet cluster. Subsequently, we calculated one-way ANOVAs on these averaged cluster values to further identify which specific cue types differed significantly and therefore caused the emergence of a significant cluster.

Analogous to the analysis of EEG data, we analyzed whether behavioral data in the induction tasks differed between the four cue types by calculating a one-way ANOVA on mean response times (RTs) and error rates (ERs) in the induction tasks. We chose to analyze induction task performance (collapsed across task sets), as analyzed EEG data reflects processing involved in preparation for induction tasks and induction task performance therefore should reflect the consequences of this preparatory processing. Furthermore, to assess whether task preparation demands indexed by EEGs predicts task performance, we correlated mean RTs and ERs in induction tasks with mean voltage and power obtained from significant ERP/wavelet clusters as described above, first across all cue types and second, within each cue type separately. We performed these correlations analyses also separately per cue type, as if there would be a non-linear relationship in EEG activity between cue types, this would probably result in a skewed correlation across cue types. For all post hoc tests and correlation analyses, we corrected for multiple testing using the Holm method.

## Results

### Behavioral data in induction tasks

**Fig. 2:**
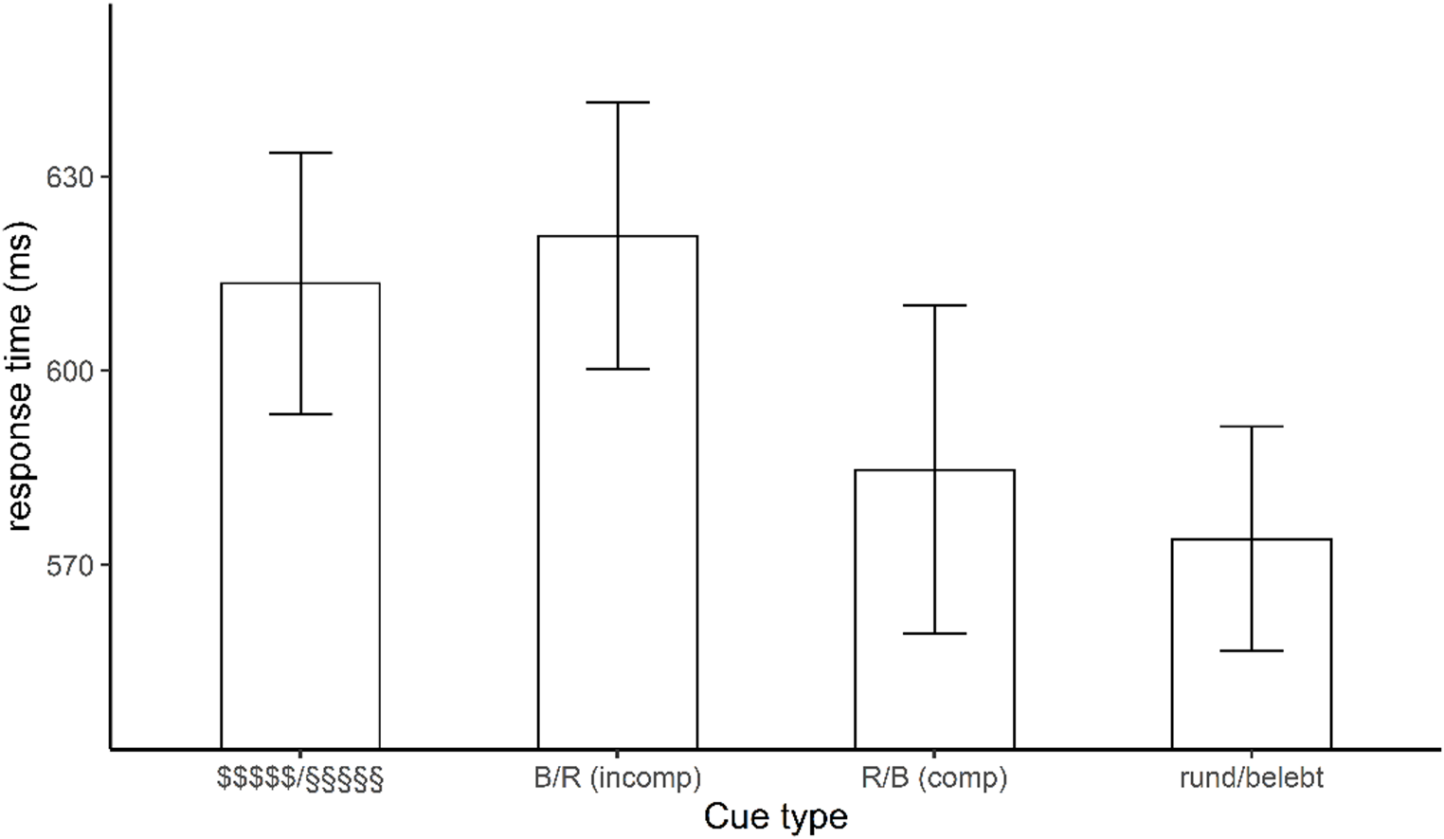
Response times in ms in induction tasks, separately for the different cue types

A one-way ANOVA on mean RTs in induction tasks with the factor cue type showed no significant RT differences between the four different cue conditions, *F*(3, 97) = 1.12, *p* = 0.346. While there were no significant differences, RTs for supposedly less transparent cues were descriptively slower (mean RT [$$$$$/§§§§§] = 613.7ms, mean RT [B/R (incomp)] = 621.2ms) compared to the supposedly more transparent cues (mean RT [R/B (comp)] = 585.1ms, mean RT [rund/belebt] = 574.2ms), see Fig. 2. For ERs, there was no significant effect of cue type as well, *F*(3, 97) = 0.58, *p* = 0.630. On a descriptive level, the highest ER was observed for the B/R (incomp) cue group (mean ER = 0.038), while mean ER was comparable for the other cue types ($$$$$/§§§§§ = 0.031, R/B [comp] = 0.030, rund/belebt = 0.030).

### Event related potentials

**Fig. 3:**
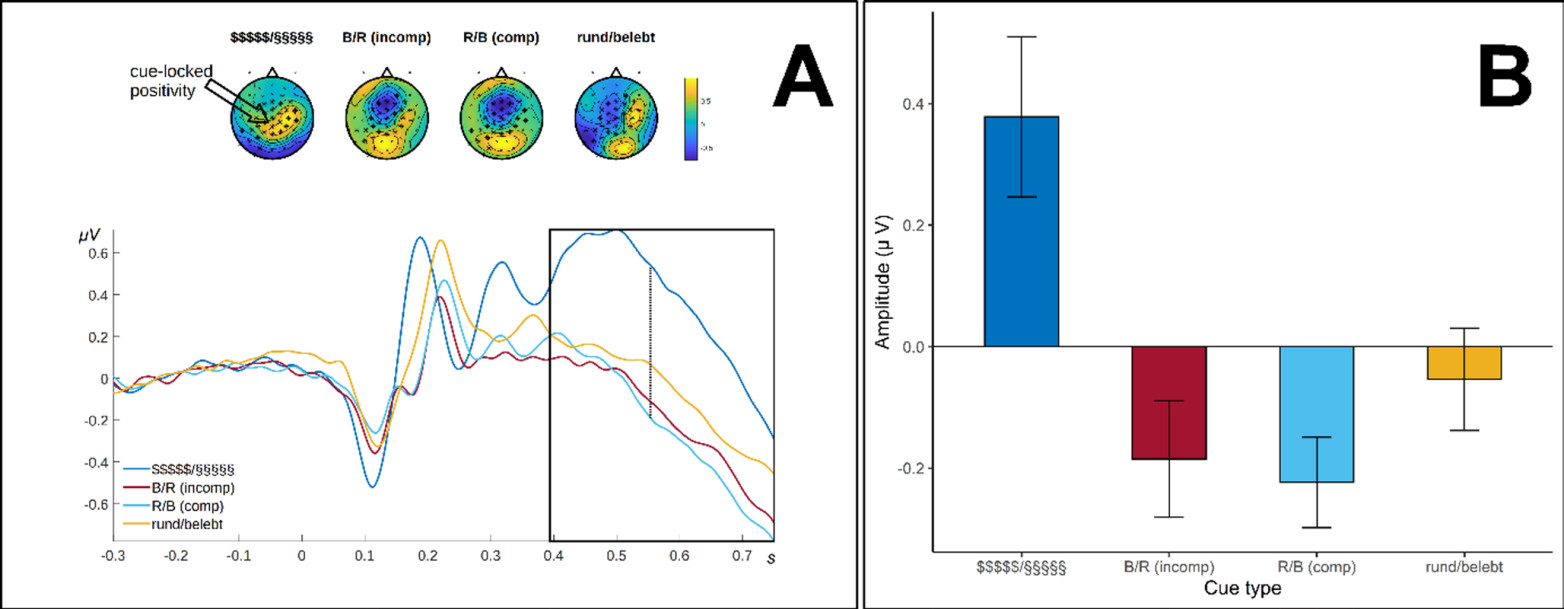
Cluster 1 for the analysis of ERPs. Panel *A* shows the result of the cluster-based permutation test comparing ERPs between all four different cue types. Topographical illustrations at the top of the panel represent the activity distribution across the scalp averaged across all time points included in the cluster. Electrodes included in the cluster are marked by an asterisk. The lower panel depicts grand-averaged ERPs for all four cue types averaged across all electrodes included in the cluster. Cue onset is at x = 0 s, cue offset at x = 0.75 s. The box indicates the temporal extents of the cluster, while the dotted vertical line shows the peak. Panel *B* shows the average voltage of this cluster separately for the different cue types, which was extracted from the cluster shown in panel A by averaging across all time points and electrodes included in the cluster

A summary of all relevant clusters obtained by CBPT of ERP and wavelet data is shown in Table 2. For ERP data, CBPT revealed two significant clusters. The first cluster ranged from 394ms relative to cue onset to cue offset and included a broad range of electrodes including central, parietal, frontal, as well as left occipital and left occipito- and tempo-parietal regions. As can be seen in Fig. 3A, this cluster reflected a cue-locked positivity component, which was increased for the symbol cues ($$$$$/§§§§§).

An ANOVA on the average voltage in this cluster revealed a significant effect of cue type, *F*(3, 97) = 7.75, *p* < 0.001, $$\:{\eta\:}^{2}\:$$= 0.193. Post Hoc tests (Holm-corrected for multiple comparisons) revealed that the symbol cues ($$$$$/§§§§§, compare Fig. 3B) significantly differed from all other cue types (all *p*s < 0.013), while the three other cue types did not differ significantly from each other (all *p*s > 0.678).

**Table 2:** Summary of all clusters for the comparison of all four cue types

	Cluster	Statistic	*P*-value	Latency	Frequencies	Electrodes
ERP	1	19725.7	< 0.001	394–750ms	-	P9, O9, O1, P7, TP7, P5, PO3, P1, CP3, C3, FC3, F1, Fz, FCz, FC1, CP1, Cz, AF4, F6, FC6, C6, Pz, P2, CP4, C4, FC4, F2, FC2, CP2, CPz
	2	2046.0	0.032	166–202ms	-	T7, FT7, AF3, F5, FC5, C5, P1, CP3, C3, FC3, F1, Fz, FCz, FC1, CP1, Cz, F6, FC6, Pz, P2, CP4, C4, FC4, F2, FC2, CP2, CPz
Wavelets	1	102237.3	0.009	0–620ms	4.4–9.3 Hz	F9, FT9, P9, O9, O1, P7, TP7, T7, FT7, AF7, AF3, F5, FC5, C5, PO3, PO1, P1, CP3, C3, FC3, F1, Fz, FCz, FC1, CP1, Cz, P10, O10, Iz, Oz, O2, P8, TP8, P6, PO4, PO2, Pz, P2, F2, FC2, CP2, CPzC4, FC4, F2, FC2, CP2, CPz

**Fig. 4:**
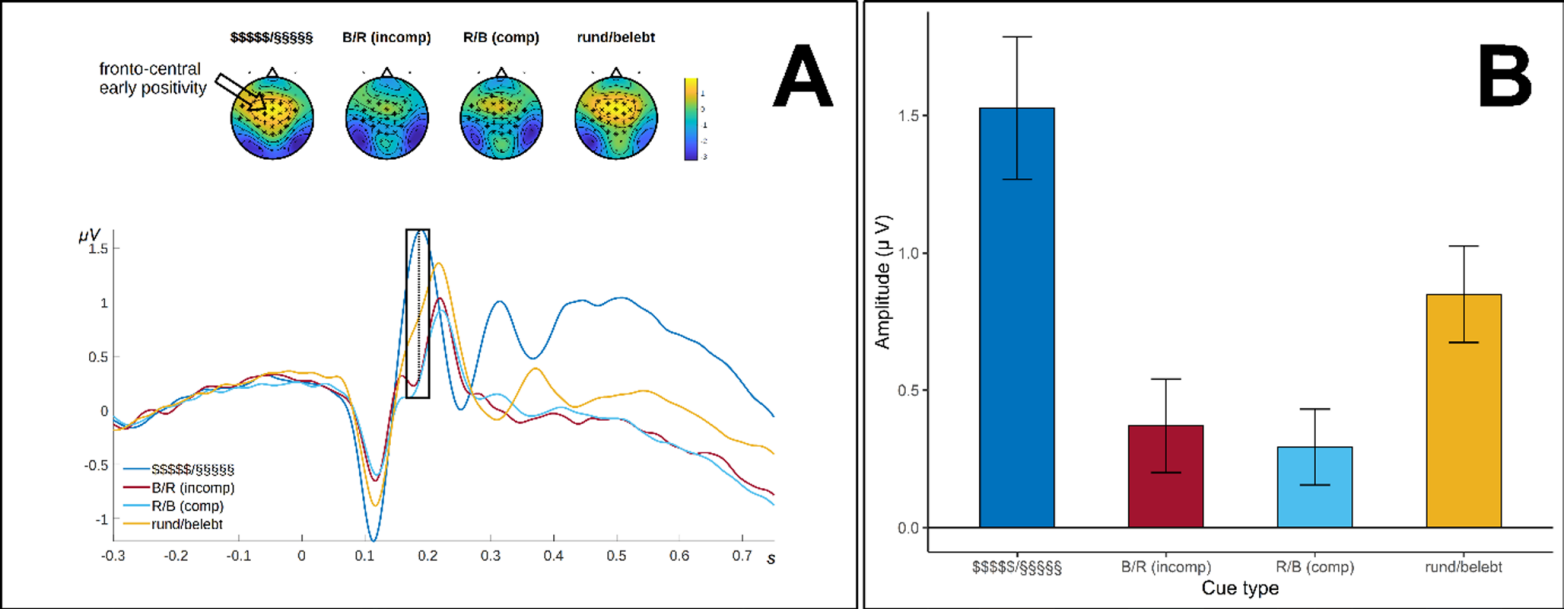
Cluster 2 for the analysis of ERPs. For a detailed description, see Fig. 3

The second significant cluster obtained by CBPT of ERP data is shown in Fig. 4. This cluster ranged from 166ms to 202ms relative to cue onset. Electrodes from central, parietal, frontal, as well as left temporal, fronto-temporal and anterior-frontal regions were included (see Fig. 4A). This cluster can probably be interpreted as showing an early P2 component (Clark and Hillyard 1996; Luck and Hillyard 1994; Potts 2004), as the visual P1 is usually observed over occipital areas (Clark and Hillyard 1996; Mangun and Hillyard 1991), and the latency of this component lies somehow between the P1 and P2 (but corresponds roughly with the N1, which is typically observed over occipital areas, Vogel and Luck 2000).

A corresponding ANOVA on the average voltage for the four different cue types in cluster 2 was significant, *F*(3, 97) = 8.91, *p* < 0.001, $$\:{\eta\:}^{2}$$ = 0.216. Post-hoc tests (Holm-corrected) revealed that the symbol cues ($$$$$/§§§§§, compare Fig. 4B) significantly differed from the letter cues (R/B [comp], B/R [incomp]; both *p*s < 0.001), while the comparison with the word cues (rund/belebt) did not reach the (corrected) significance level, *p* = 0.060. The comparisons among the other three cue types did not reveal any significant difference, all *p*s > 0.122.

### Wavelets

**Fig. 5:**
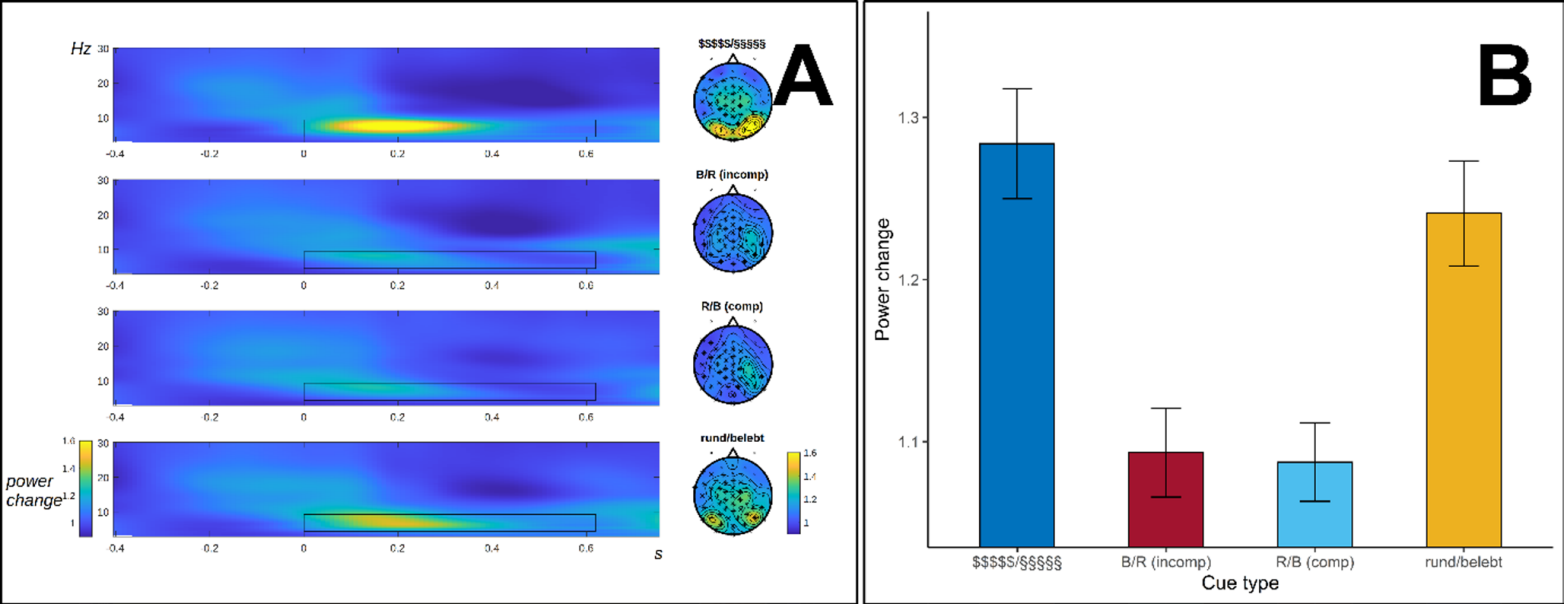
Cluster 1 for the analysis of wavelets. Panel *A* shows the result of the cluster-based permutation test comparing wavelets between all four different cue types. The left side of panel *A* depicts grand-averaged power values for all four cue types averaged across all electrodes included in the cluster. Cue onset is at x = 0 s, cue offset at x = 0.75 s. The box represents the temporal extents as well as the included frequencies of the cluster. Topographical illustrations at the right side of panel *A* represent the distribution of power activity across the scalp averaged across all time points and frequencies included in the cluster, with the topographical plot for each cue type being aside the respective time-frequency representation of this cue type shown in the left side. Electrodes included in the cluster are marked by an asterisk. Panel B shows the average power change relative to the baseline of this cluster separately for the different cue types, which was extracted from the cluster shown in panel A by averaging across all time points, electrodes and frequencies included in the cluster

CBPT of wavelet data revealed one cluster, where a significant difference between the four cue types was observed (see also Table 2). It included frequencies in the theta band (4–9 Hz) and lasted from the beginning to almost the end of the analyzed time interval (0–620ms relative to cue onset). It included regions across almost the whole scalp, including central, parietal, frontal and occipital locations. Furthermore, it included left temporal, left fronto-temporal and left anterior-frontal regions. Figure 5A shows an overview of this cluster.

An ANOVA on the clusters’ averaged power revealed a significant effect of cue type, *F*(3, 97) = 11.68, *p* < 0.001, $$\:{\eta\:}^{2}$$ = 0.265, see Fig. 5B. Post-hoc tests (Holm-corrected) revealed the symbol cues $$$$$/§§§§§ to differ significantly from the letter cues (R/B [comp], B/R [incomp]; both *p*s < 0.001). Moreover, the word cues (rund/belebt) significantly differed from both letter cues, both *p*s < 0.003. The comparisons between letter cues as well as the comparison between symbol and word cues did not reveal significant differences (both *p*s > 0.634).

### Correlation of EEG data and behavioral data

**Fig. 6:**
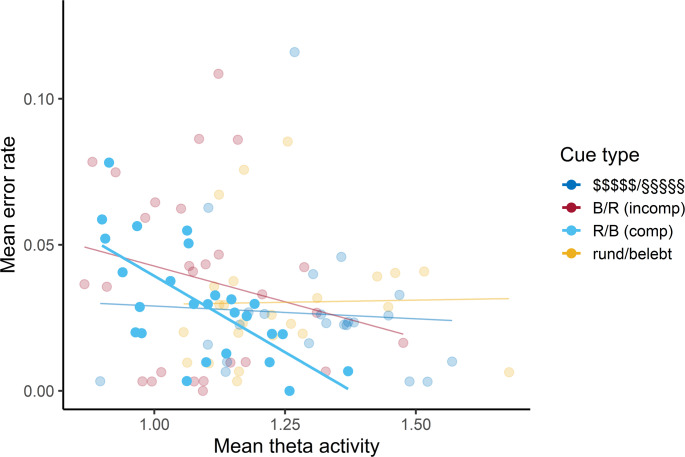
Scatter plot for the relationship between mean theta activity obtained in the wavelet cluster and mean error rate, separately for the four cue types. The group with the compatible letter cues (R/B [comp]) is shown with higher opacity, as the correlation in this group reached significance, *r* = -0.67, *p* = 0.001

To assess whether task preparation indexed by ERPs and oscillatory electrical brain activity predicted actual task performance, we correlated mean voltage and power in the obtained clusters per subject with respective mean RTs and ERs in induction tasks of these subjects, first collapsed across cue types and subsequently separately per cue type. Regarding the analyses collapsed across cue types, for RTs (all |*r*|s < 0.14, all *p*s > 0.566) there was no significant (Holm-corrected) correlation with neither ERPs nor oscillatory activity. For ERs, the correlation with oscillatory activity just missed the corrected significance level, *r* = -0.23, *p* = 0.057, but reflected a lower error rate for participants with a more pronounced theta activity in the cue interval. For the analysis of ERPs across cue types, no significant correlation could be observed (both |*r*|s < 0.04, all *p*s > 0.999). However, regarding the separate analyses for the different cue types, there was one significant correlation between oscillatory activity and ERs, *r* = -0.67, *p* = 0.001, in the group with compatible letter cues (R/B [comp]). This correlation reflected fewer errors for participants with a higher increase of theta activity in the cue interval. For a visualization of this correlation (among the other, non-significant correlations of mean ERs and theta power for the other cue types), see Fig. 6. The correlations of RTs/ERs with ERPs and oscillatory electrical brain activity in the other three cue type groups were not significant after Holm correction, all |*r*|s < 0.33, all *p*s > 0.421.

## Discussion

The present study investigated preparatory electrophysiological activity in response to task cues, analyzing both ERP and oscillatory brain activity. We hypothesized that engagement in task preparation is reflected in the cue-locked positivity and oscillatory brain activity in the theta band and would accordingly differ across cues with a varying level of task cue transparency. In line with this hypothesis, we observed that a cue-locked positivity ERP component was increased for symbol cues lacking any apparent relation with the cued task. Unexpectedly, the analysis of ERP data revealed a positive deflection probably showing an early P2 to be more pronounced for the symbol and word cues. Furthermore, the symbol and word cues elicited more theta power compared to the letter cues. As these two types of cues were considered to be the least and most transparent ones, respectively, these findings indicate further factors beyond task cue transparency such as cue length to influence cue processing. Before discussing the implications of the present work regarding the engagement into task preparation in the current study, as well as optimal cue design in general, we will outline which processes may have contributed to the obtained differences between cue types reflected by the observed significant clusters.

### Differences in event-related activity between cue types

CBPTs of ERPs revealed a cue-locked positivity component to be altered by cue type (see Fig. 3A), showing a similar latency and topography as the switch positivity (Kieffaber and Hetrick 2005). Previous work however indicated this component not only to be modulated by task switches vs. repetitions in task switching, but also more generally by task demands (Jost et al. 2008; Karayanidis et al. 2009; Sinai et al. 2007). Accordingly, this cue-locked positivity may reflect a more general index of engagement in task preparation (Jamadar et al. 2015). Contributing to this above-mentioned work, the present study revealed the cue type as an additional influence on the electrophysiological correlates of task preparation demands.

Separate analyses for the different cue types revealed the increased cue-locked positivity within the identified cluster to be confined to the symbol cues ($$$$$/§§§§§), while for the other cue types the size of the cue-locked positivity was smaller and similar (see Fig. 3B). A larger cue-locked positivity is in line with accounts assuming a more challenging task preparation process, if cues are less transparent, i.e., if it is supposedly more difficult to retrieve the relevant task set from memory (Jost et al. 2013; Logan and Schneider 2006). Hence, task preparation seemed to be especially demanding for the arbitrary symbol cues, for which any apparent relation between cue and decision categories lacked. For the other cue types, for which cues and decision categories could be at least linked by a mediator (Logan and Schneider 2006), retrieval of task sets appeared to be less demanding. In line with this observation, it should be added that the symbol cues supposedly were the most difficult ones to discriminate. For the other cue types, participants had to discriminate between the letters R and B and the words “rund” (engl. “round”) and “belebt” (engl. “living”), which was certainly easier than discriminating between two symbol strings ($$$$$ and §§§§§). However, the topography of the observed cluster did not include occipital areas, rendering it plausible that the effect on the cue-locked positivity component mainly represents increased cognitive rather than visual engagement.

Although not predicted, the second significant cluster revealed by CBPT analysis on ERP data may have reflected visual and attentional demands associated with visual complexity of cues. In this cluster, a positive deflection around 150 to 200ms at fronto-central electrodes was most pronounced for the symbol ($$$$$/§§§§§) and word cues (rund/belebt). In contrast, for the two letter cue conditions (R/B [comp], B/R [incomp]), this component was comparably smaller (compare Fig. 4). Due to its fronto-central location, this component can probably be interpreted as reflecting an early P2 component (Clark and Hillyard 1996; Luck and Hillyard 1994; Potts 2004), as the P2 mostly starts around 200ms post-stimulus (temporally, this positive potential overlapped with the occipital N1, cf.; Clark and Hillyard 1996; Vogel and Luck 2000). In line with its discussed relevance for the analysis of stimulus features (Luck and Hillyard 1994; Potts 2004), this increased positive potential could reflect the higher visual complexity of character string cues (symbol and word cues). Compared to cues consisting of only one letter, it may be more demanding, and more attention may be required to retrieve the correct task set, if cues are more complex and the pair of cues used for the two task sets are thus more difficult to distinguish. Obviously, visual demands are related to the amount and complexity of visual information which needs to be processed. Moreover, these demands were especially increased for the symbol cues, in which cues for the two tasks consisted of relatively similar symbols ($ vs. §), while the comparison between word cues (rund/belebt) and letter cues did not cross the corrected significance level as indicated by corresponding Post Hoc tests.

To summarize, analysis of ERP data revealed two components to be affected by the type of task cue, a late cue-locked positivity component presumably reflecting demands associated with activating/preparing a task set, and an earlier positive component (probably reflecting an early P2), reflecting visual processing demands according to the complexity of visual information comprising the cue.

### Differences in oscillatory activity between cue types

Besides these ERP effects, oscillatory electrical activity was affected by cue type: Theta power differed between cue types, for almost the whole cue period (compare Fig. 5A). In general, the theta band was discussed as an index for cognitive control processes, in particular the updating of such processes, for instance the increased recruitment of cognitive control following an error trial (for a review, see: Cavanagh and Frank 2014). In task switching, more specifically, increased theta power in switch than repeat trials was thought to reflect cognitive demands for task set reconfiguration (Cooper et al. 2017; López et al. 2019). In the present work, we assessed theta power in the cue interval relative to a task-unrelated fixation cross period (relative baseline correction), and oscillatory power therefore reflected the engagement into cognitive control processes relative to that baseline. When comparing theta power across the different cue types, it was increased for the symbol ($$$$$/§§§§§) and word cues (rund/belebt) compared to the letter cues (R/B [comp] and B/R [incomp]), see Fig. 5B. Thus, the processing of task cues appeared especially demanding for the longer character string cues including symbols and words. While elevated processing demands for the symbol cues were not surprising and were in accordance with the hypothesized low transparency of these cues, as well as the results of the ERP analyses, the increased theta power for the word cues was not predicted. These cues were considered to be the most transparent ones, and thus, task set activation should have been facilitated for these cues. We consider two explanations conceivable for the increased theta power for word cues. First, similar to the second ERP cluster, visual demands may have contributed to this finding. In contrast to the letter cues, it is plausible that some “reading”-related processes are involved in processing the word cues, which may have posed cognitive demands, especially in comparison to the baseline period, where a fixation cross (“+”-symbol) was presented. Second, in line with accounts relating the theta band to updating processes, in particular the reconfiguration of task sets (Cooper et al. 2017; López et al. 2019), such a reconfiguration may have been especially demanding for the most transparent cues. Increased inhibition effects were observed for so-called dominant task sets with a strong mental representation (Jost et al. 2017; Kiefer et al. 2019). Similarly, the notion of asymmetric switch costs indicates that dominant/strongly represented task sets interfere more with other, competing tasks (Allport et al. 1994; Kiesel et al. 2010). Thus, reconfiguring task sets may have been especially demanding for the word cues, as these cues were the most transparent ones and cued task sets therefore had a strong mental representation. Accordingly, they may have competed to a higher degree with the task set of the non-cued task. However, as suggested by the cue-locked positivity and behavioral performance, this conflict might have been resolved early (compare also Fig. 5A, which suggests the theta increase to peak around 200ms), resulting in a decent level of task set activation upon cue offset. In a similar vein, switch costs in task switching are usually lower for more transparent cues, indicating that at least during task processing following the cue, a decent level of task set activation is reached for transparent cues (Gade and Steinhauser 2020; Grange and Houghton 2010; Jost et al. 2013). Accordingly, while the word cues may have been initially demanding, these demands appeared to be sufficiently overcome during the 750ms cue period.

### Effects of cue type on task preparation

To integrate the results of the ERP and wavelet analyses, the largest cognitive demands for task preparation were consistently observed for the symbol cues, which lacked any apparent relation with the cued task set. For the other types of cues, processing demands were lower, despite of increased visual demands and probably demands to overcome the competition between concurring task sets for the word cues (increased early positivity, increased theta activity). The overall highest preparation demands for the least transparent cues (symbol cues, $$$$$/§§§§§) are in line with previous behavioral findings of increased costs in task switching for less transparent cues (Gade and Koch 2014; Gade and Steinhauser 2020; Grange and Houghton 2010; Houghton et al. 2009; Jost et al. 2013). Extending this previous research, the present work could demonstrate effects of task cue transparency to be already observable during the task preparation period, while previous observed effects on response times and error rates could only reflect influences of task cue transparency in terms of a result of task execution. In contrast, increased demands for the word cues (rund/belebt), which were initially considered to be the most transparent ones, indicated that other factors beyond task cue transparency influence the processing of task cues (for possible consequences concerning the choice of task cues, see the next section). Regarding the letter cues, any pronounced differences in EEG activity lacked between compatible and incompatible cues. Thus, the relation between cue and decision categories via a mediator for incompatible cues (e.g. cue “B” → “belebt” → mediator “use opposing task” → “rund”; cf. Logan and Schneider 2006) appeared to result in similar task preparation demands compared to the direct link via cuing the first letter of the decision category (cue “B” → “belebt”). Probably, participants relied on such strategies to facilitate cue processing. For the symbol cues, for which such strategies likely were not available, a decent task set activity could nevertheless be accomplished during the cue interval. As presumably sufficient time for task preparation was available (750ms cue interval), task sets could be successfully prepared in the cue interval by presumably employing larger cognitive demands, as indicated by comparable effects of task preparation on subsequent priming compared to the other cue types (Berger et al. 2022).

In line with this reasoning, there was no main effect of cue type on the RT in induction tasks, indicating a similar task processing speed between task cue transparency conditions. While descriptive differences between cue types were present (compare Fig. 2), they were only small and did not reach significance. Note, however, that in later studies in which we included the two cue types R/B (comp) and B/R (incomp) in a task switching design, we observed participants in the latter cue condition to respond significantly slower (up to 125ms, see; Berger et al. 2024; Berger and Kiefer 2025). In contrast to the present one, in which trials were initiated in a self-paced fashion, in these more recent studies there was a continuous flow of trials, which might have increased switch and preparation demands and thus increased cue type effects. Thus, the present work might have underestimated the impact of cue type in contrast to what would have been expected in a typical task switching context. Moreover, the comparable performance between cue types in the present study may also be the reason, why correlations between EEG measures of task preparation and behavioral data (RT, ER) mostly lacked. Only within the group of compatible letter cues (R/B [comp]), a significant negative correlation between theta activity and ER in induction tasks was observed (see Fig. 6). However, this correlation was large and indicated that the more theta was employed during cue processing, the fewer errors were conducted when the cued task had to be performed later. This relation was also present in the correlation analysis across cue types (however not significant, *p* = 0.057), but appeared to be mainly driven by the R/B (comp) and partially by the B/R (incomp) cues. Interestingly, this relation was entirely absent for symbol and word cues. Probably, a relation between EEG activity and behavior could only be observed for letter cues, in particular for the compatible ones, as it appeared to be the overall easiest one to recognize, with the most straightforward cue-task linkage. Furthermore, across all participants, both compatible and incompatible letter cues elicited less theta power than symbol and word cues. Thus, an interindividual different engagement into cue processing reflected by theta activity could translate into differences in behavioral performance, while this relation was concealed in the other conditions, possibly due to generally higher theta activity. We will elaborate on this point a bit more, when discussing which type of cue may be optimal for cuing a task in the next section.

### Recommendations regarding the optimal type of cue for task preparation

When integrating all EEG analyses, R/B (comp) appeared to be the easiest type of cue. It had (numerically) the lowest cue-locked positivity, the smallest early P2 and the smallest theta increase compared to the baseline. For the symbol strings ($$$$$/§§§§§), all these measures reflecting demands of cue processing were increased, while for the word cues (rund/belebt) the latter two were larger compared to R/B (comp). Only the incompatible letter cues (B/R [incomp]) showed a similar EEG activity compared to the compatible ones. However, for these cues, RTs were descriptively slower, and ERs were descriptively increased. Hence, when conjointly considering EEG and behavioral measures, R/B (comp) appeared to be (at least descriptively) the type of cue associated with the lowest processing demands and best performance. For the cues comprising of character strings (symbol and word cues) visual demands as indexed by the ERP effect in the early P2 time window and cognitive demands as indexed by theta activity were higher. For the incompatible letter cues, the linkage between cue and decision category could only be achieved by a mediator, which might have caused confusion for some participants.^3^

Accordingly, based on the present findings, we would recommend using letter cues with a straightforward relation to the cued task (for example, by using the first letter of the task/decision category). Such cues showed a similar ease in activating the cued task set (reflected by e.g. the cue-locked positivity) compared to the supposedly more transparent word cues, while keeping visual demands and cognitive control demands low. Such low visual and cognitive control demands may have an even greater influence, if a shorter cue duration compared to the 750ms used in the present study is chosen.

## Conclusion

The present electrophysiological results suggest that engagement in proactive control in response to task cues differs according to the type of task cue. Thus, our research indicates that properties of the task cue influence cue-induced task preparation processes, which may translate into subsequent behavior. For facilitating the cue-induced task set activation process, it is crucial to choose an appropriate cue. For cues, which lacked any apparent connection with the cued task, cognitive demands reflected by a cue-locked positivity component were increased. Furthermore, visual demands reflected by an early P2 component and theta activity were increased for character string cues, indicating that cues comprising of many symbols/letters may be more demanding than choosing only a single letter. Hence, a simple letter cue with a straightforward relation to the cued task appeared to pose the least demands on cue processing. To conclude, as the electrophysiological correlates of task preparation demands differed between the type of task cue it is crucial to consider the type of task cue in studies investigating proactive control processes elicited by task cues.

## Footnotes

1 The segmented time window was slightly larger relative to cue offset for task cue-only trials to ensure that the same length following the task stimulus, which was presented after the cue, is included for the sake of comparability. The task stimulus in task cue-only trials was the LDT target, which was preceded by a 167ms prime interval. Thus, the segmented time interval was 167ms larger for task cue-only trials.

2 Given the specifications of the wavelet analysis, the half wavelet duration was 279ms for 4 Hz, 248ms for 4.5 Hz, and 223ms for 5 Hz. As we were interested in the theta frequency band, we tried to avoid any leakage of cue-related oscillatory activity into the baseline period starting at these frequencies. Given the chosen baseline window, an influence of cue activity into the baseline window could be precluded for all frequencies higher than 4.5 Hz.

3 To further corroborate this reasoning, we analyzed performance in the practice session. In a practice session comprising of several blocks preceding the experimental session, participants could repeat practice blocks until they achieved a decent performance (for details, see Berger et al. 2022). In two of these blocks, they had to perform both induction tasks together, and therefore had to retrieve the required task set from the cue. Participants in the B/R (incomp) group repeated these blocks the most often; mean number of practiced blocks: $$$$$/§§§§§ = 2.96, B/R (incomp) = 3.33, R/B (comp) = 2.54, rund/belebt = 2.38. Thus, the incompatible cue-task relation led to (initial) confusing, therefore requiring participants to practice this assignment more often. A corresponding ANOVA with the factor cue type was significant, *F*(3, 97) = 5.88, *p* < 0.001, *η*^2^ = 0.154.
